# From Orchard to Wellness: Unveiling the Health Effects of Sweet Cherry Nutrients

**DOI:** 10.3390/nu16213660

**Published:** 2024-10-28

**Authors:** Berta Gonçalves, Alfredo Aires, Ivo Oliveira, Miguel Baltazar, Fernanda Cosme, Sílvia Afonso, Teresa Pinto, Maria Rosário Anjos, António Inês, Maria Cristina Morais, Alice Vilela, Ana Paula Silva

**Affiliations:** 1 Centre for the Research and Technology of Agro-Environmental and Biological Sciences (CITAB), Institute for Innovation, Capacity Building and Sustainability of Agri-Food Production (Inov4Agro), University of Trás-of-Montes e Alto Douro, Quinta de Prados, 5000-801 Vila Real, Portugal; alfredoa@utad.pt (A.A.); ivo.vaz.oliveira@utad.pt (I.O.); migueladbaltazar@gmail.com (M.B.); safonso@utad.pt (S.A.); tpinto@utad.pt (T.P.); ranjos@utad.pt (M.R.A.); mariacristina.morais@gmail.com (M.C.M.); asilva@utad.pt (A.P.S.); 2 Chemistry Research Centre-Vila Real (CQ-VR), University of Trás-of-Montes e Alto Douro, Quinta de Prados, 5000-801 Vila Real, Portugal; fcosme@utad.pt (F.C.); aines@utad.pt (A.I.); avimoura@utad.pt (A.V.)

**Keywords:** bioactive compounds, health effects, *Prunus avium* L., sweet cherry nutrients

## Abstract

This review paper explores the multifaceted relationship between sweet cherry nutrients and human health, aiming to uncover the comprehensive impact of these bioactive compounds from orchard to wellness. Furthermore, it highlights how advanced crop techniques can be pivotal in optimizing these beneficial compounds. Synthesizing existing literature, the paper examines the diverse bioactive nutrients in sweet cherries, including antioxidants, polyphenols, vitamins, and minerals, and elucidating their mechanisms of action and potential health benefits. From antioxidant properties to anti-inflammatory effects, the paper elucidates how these nutrients may mitigate chronic diseases such as cardiovascular disorders, diabetes, and neurodegenerative conditions. Additionally, it explores their role in promoting gastrointestinal health, enhancing exercise recovery, and modulating sleep patterns. The review discusses emerging research on the potential anti-cancer properties of sweet cherry compounds, highlighting their promising role in cancer prevention and treatment. Furthermore, it delves into the impact of sweet cherry consumption on metabolic health, weight management, and skin health. By providing a comprehensive overview of the current understanding of sweet cherry nutrients and their health effects, this paper offers valuable insights for researchers, healthcare professionals, and consumers interested in utilizing nature’s bounty for holistic wellness.

## 1. Introduction to Sweet Cherry Nutrients and Human Health

Sweet cherries (*Prunus avium* L.) are nutritious fruits recognized for their rich composition of beneficial compounds. They are an excellent source of vitamins, minerals, fiber, and polyphenols, which are plant compounds known for their antioxidant and anti-inflammatory properties. Research indicates that the polyphenol content can vary among cherry cultivars, but all cherries are generally classified as polyphenol-rich foods, contributing to their health benefits [1,2,3,4].

In addition to polyphenols, sweet cherries contain carotenoid pigments like β-carotene, as well as vitamin C, which enhance their antioxidant capacity [5]. These compounds play a crucial role in combating oxidative stress, a condition linked to various chronic diseases such as cancer, cardiovascular diseases, and diabetes. Recent studies have highlighted the health-promoting properties of sweet cherries, emphasizing their potential to prevent and manage these conditions through their rich bioactive compound profile.

The nutrient-dense profile of sweet cherries makes them a valuable addition to the diet [6]. One cup of sweet cherries (150) provides significant amounts of essential nutrients, including vitamin C, potassium, and dietary fiber, all supporting overall health [7]. The fiber content in cherries aids in digestive health and promotes beneficial gut bacteria, while their low glycemic index makes them a preferable fruit choice for blood sugar management [3].

Overall, sweet cherries enrich the diet with essential vitamins and minerals and offer a range of health benefits due to their high content of antioxidants and anti-inflammatory compounds. This makes them a commendable choice for those seeking to enhance their nutritional intake and support their health.

This review aims to comprehensively examine the relationship between the nutrients found in sweet cherries and human health, from cultivation practices to their bioactive effects. Additionally, it seeks to provide valuable insights for researchers, healthcare professionals, and consumers on the health-promoting potential of sweet cherries.

## 2. Exploring the Bioactive Compounds in Sweet Cherries

**Figure 1:**
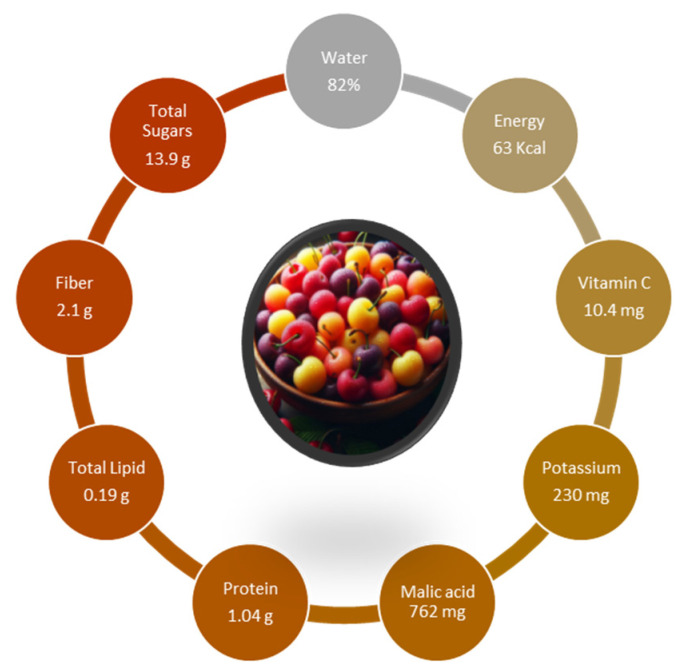
Nutritional composition of cherries per 100 g. Adapted from [7].

Figure 1 illustrates the nutritional composition of cherries per 100 g, highlighting key components such as water content (82%), energy (63 kcal), vitamin C (10.4 mg), potassium (230 mg), and fiber (2.1 g) [7].

### 2.1. Polyphenols, Vitamins, and Minerals

The polyphenol composition of sweet cherries is diverse, comprising various compounds, including anthocyanins, flavonols, hydroxycinnamic acids, and flavan-3-ols [1]. Each polyphenol group contributes to the fruit’s nutritional value and health-promoting effects [2]. Milea et al. [8] reported that sweet cherries are a rich source of phenolics, with an average total phenolic concentration of 150 mg/100 g of fresh weight (FW), of which 60–74% are hydroxycinnamic acid derivatives, anthocyanins, and flavanols, mainly flavan-3-ols. Also, Gonçalves et al. [9] identified 46 phenolic compounds in different sweet cherry varieties ranging from 10.6 to 508.5 mg/100 g dry weight (DW), and total anthocyanin content ranging from 0.46 to 427.6 mg/100 g DW [9]. Another important group of polyphenols in sweet cherries are flavonols, with quercetin and kaempferolbeing the most significant [10]. Sweet cherries also contain high levels of hydroxycinnamic acids, including neochlorogenic acid and chlorogenic acid [1,11,12]. Neochlorogenic acid is effective at scavenging free radicals and protecting cells from oxidative damage, while chlorogenic acid is known for its potential to regulate blood sugar levels and improve glucose metabolism [13]. Epicatechin and catechin, which belong to the flavan-3-ols class, are other key polyphenols in sweet cherries. Research indicated that catechin and epicatechin, two major flavan-3-ols in sweet cherries, can enhance blood flow and reduce the risk of atherosclerosis [14]. Catechin exhibits strong antioxidant activity [15], and both compounds appear to support heart health and circulation, contributing to the prevention of cardiovascular diseases [16].

Sweet cherries are also rich in vitamins, particularly C, A, and B [7]. Vitamin C, also known as ascorbic acid, is one of the most abundant vitamins in sweet cherries, as reported by Bastos et al. [17]. This water-soluble vitamin is a powerful antioxidant that helps protect cells from damage caused by free radicals. Vitamin C is crucial for collagen synthesis, a protein that supports the structure of skin, blood vessels, and connective tissues. It also aids in iron absorption from plant-based foods, supports the immune system, and promotes wound healing. Vitamin A, in the form of β-carotene, a precursor of vitamin A, is essential for maintaining healthy vision, particularly in low-light conditions, supporting immune function, and promoting healthy skin and mucous membranes [18]. Sweet cherries are also known as a good source of B vitamins, including B6 (pyridoxine), B9 (folate), and B1 (thiamine) [7].

Regarding mineral composition, sweet cherries contain calcium (14 mg/100 g), magnesium (10 mg/100 g), phosphorus (20 mg/100 g), and potassium (200 mg/100 g) [4]. These minerals are essential dietary constituents that play a vital role in the structural components of the human body, such as bones and teeth, and are involved in several metabolic processes, including normal cell function, energy production, protein synthesis regulation of blood pressure, the guarantee of muscle contraction, nerve signal transmission, and the proper functioning of the kidneys and liver, among other vital organs [19].

### 2.2. Sugars, Organic Acids, Proteins, and Fiber

Sweet cherry fruits are highly appreciated by consumers not only for their content of bioactive compounds but also for their well-balanced taste and aroma. This balance is due to their composition of sugars, which contribute to sweetness; organic acids, which are key to their sourness; and proteins [20]. The sugar content in sweet cherries is relatively low, ranging from 11 to 26% [9,21,22], while organic acids vary from 3.67 to 8.66 g/kg FW [9,22] and the protein content is usually between 0.8 and 1.4% [17,23]. Sugars in sweet cherries are divided into monosaccharides, oligosaccharides, and polysaccharides, with the first two groups being the primary contributors to the fruit’s sweet taste [24]. The most abundant sugars in sweet cherry are monosaccharides or simple sugars (for example, glucose, fructose, and galactose). Glucose and fructose represent more than 90% of total sweet cherry sugars [25], with values varying from 6.0 to 10.0 g/100 g of fruit for glucose and from 5.3 to 10.1 g/100 g for fructose, depending on several factors, including cultivar [17,26,27,28]. Galactose is also referenced to be present, but in smaller amounts than other monosaccharides, around 0.59 g/100 g [23], and xylose has also been reported, but in minimal quantities (0.015 to 2.26 g/100 g) [11]. For disaccharides, the most abundant in sweet cherry are sucrose and maltose, with contents of around 0.12–0.15 g/100 g [23]. However, sucrose content can vary from 0.36 to 1.2 g/100 g, depending on the cultivar [27,28]. Sweet cherries contain sugar alcohols, mainly sorbitol and ribitol [11]. The latter’s content varies between 0.058 g/100 g and 0.146 g/100 g, depending on the cultivar [11]. Its content can change considerably for sorbitol when comparing cultivars, being as low as 0.93 g/100 g to 6.88 g/100 g [11,28,29,30]. It is vital to sorbitol levels, as it is a sugar alcohol essential for controlling weight and dental health [30], but it can also improve the taste and texture of fruits [31]. Besides the influence of cultivars on the levels of these different sugars, other aspects have to be considered, such as agronomic factors, environmental conditions, and fruit development and ripening stage [28,32,33,34]. Organic acids are a vital component of sweet cherry quality, as they are responsible for the sour taste, which contributes to the overall cherry fruit flavor [35]. The amount and balance of acids and sugars considerably affect the taste of fruit and, subsequently, influence the acceptance of consumers [24]. These compounds are essential to the metabolism, as their oxidation in the Krebs cycle provides energy for maintaining cell integrity while originating several constituents, including amino acids [36]. The organic acids in cherries are approximately 1% of the fresh edible portion [28,37]. Several organic acids have been identified in sweet cherry and usually include malic, shikimic, fumaric, quinic, and citric acids, with malic acid representing the major one, comprising over 98% of the total organic acid content [20,28,29,32]. Even so, the content of organic acids can vary significantly among cultivars [11,28,29,30,38] and can provide the quantification for other organic acids, like ascorbic acid [17,32], acetic acid [11], or tartaric acid [34]. Furthermore, in some situations, the major organic acid cannot be malic acid but rather ascorbic [39], oxalic, or tartaric acid [40].

The protein content in sweet cherries is low, usually between 0.8 and 1.4% [17,23,41], even though lower values of 0.42 or 0.58 have been recorded [17,27]. Concerning amino acids, there is little information, with early data indicating the presence of 18 essential and nonessential amino acids in sweet cherries: threonine, isoleucine, leucine, lysine, methionine, cystine, phenylalanine, tyrosine, valine, aspartic acid, glutamic acid, glycine, proline, tryptophan, and serine [7]. Novel works showed the presence of a total of 35 amino acids [27]. The content of free amino acids was, on average, about 2200 mg/100 g FW, with asparagine representing the most abundant one (over 95% of the total), followed by glutamine. Other critical amino acids include glutamic, proline, aspartic, and valine. Amino acids are essential for producing several compounds, including enzymes, hormones, antibodies, transporters, and muscle fiber in the human body [42]. Sweet cherry intake has been reported to increase the levels of tryptophan in the brain, with a subsequent increase in serotonin and melatonin in the brain and blood [43], as tryptophan can cross the blood–brain barrier and be converted into serotonin, but also increase the production of tryptamine, quinolinic acid, kynurenic acid, and coenzymes nicotinamide adenine dinucleotide (NAD) and nicotinamide adenine dinucleotide phosphate (NADP) [44].

Sweet cherries are also a good source of dietary fiber, with 1.7 to 2.4 g/100 g FW [7], which constitutes about 7% of the recommended daily intake of dietary fiber. Although sweet cherries do not have the highest fiber content among red fruits [45], their consumption can be associated with health benefits [26], including reducing the risk of gastrointestinal disorders [46] and preventing diabetes [47] and other chronic diseases [48]. The beneficial effect of eating sweet cherries can be linked to their low glycemic index (GI) due to their fiber content [4]. Soluble fiber slows sugar absorption, which helps control blood sugar levels, thereby reducing the risk of type 2 diabetes [49]. The fiber in sweet cherries also helps to reduce cholesterol levels and, thus, the risk of cardiovascular disease [26]. In addition, sweet cherries can contribute to weight loss and lower the risk of obesity due to the satiating effect of their fiber content [50]. The health benefits of sweet cherries also extend to the digestive system. The fiber content of these fruits promotes healthy digestion [48] and prevents gastrointestinal inflammation.

## 3. Factors Affecting Sweet Cherry Quality and Health-Promoting Compounds from Growth to Postharvest

Consumers, who are increasingly informed and demanding, seek foods that are attractive and rich in bioactive compounds with high nutritional value and that promote human health benefits. Sweet cherries, commonly consumed as fresh fruit, stand out as one of the ideal fruits among consumers because they possess this comprehensive set of essential and relevant characteristics, which has significantly increased their consumption [4,25,51]. The value attributed to sweet cherries is closely linked to their quality attributes, such as color, size, sweetness, firmness, flavor and aroma; the presence of a green stem; and the absence of defects, conferring substantial economic potential to this fruit [52,53,54,55].

However, due to its sensitivity to climatic fluctuations, such as excessive rainfall before harvest, this crop faces several limitations and challenges throughout its vegetative cycle. These can compromise fruit quality and, consequently, its market value [56,57]. Therefore, growers must adopt new technologies and agricultural practices to avoid significant economic losses.

Additionally, sweet cherries are a non-climacteric fruit with a high transpiration rate. They are highly perishable and have a short shelf life, complicating their export to distant markets [55,58]. This requires appropriate post-harvest procedures to preserve the physical and organoleptic qualities characteristic of this fruit.

Adopting new technologies and cultural practices, edible coatings, and storage in controlled atmospheres have shown great potential for improving sweet cherry pre- and post-harvest conditions. These methods ensure more excellent production of this fruit and enhance its quality and longevity.

### 3.1. Factors Influencing the Quality of Sweet Cherries During Growth

#### 3.1.1. Climate and Soil

Climate plays a crucial role in the growth and profitability of high-quality sweet cherry production, as cherry trees are highly sensitive to climatic variations. Most sweet cherry cultivars require a substantial number of chilling hours (over 700 h below 7.2 °C) to ensure the proper breaking of dormancy [59]. Insufficient chilling hours can disrupt the development of floral buds, resulting in poor pollen and pistil formation, which limits pollination. Optimal flowering temperatures, above 15 °C, are necessary for efficient pollination and fruit set [59,60,61]. Furthermore, fruit quality parameters such as size, weight, and soluble solid content can be enhanced in warmer years compared to cooler ones, according to a study conducted by Gonçalves et al. [1]. However, the general increase in temperatures due to climate change can alter the required chilling accumulation for cherries, affecting both their phenology and their production [62]. Additionally, temperatures above 30 °C can lead to the development of double fruits and fruit burn, rendering them unmarketable [59,63]. As the most critical and sensitive periods in the development of cherry trees are during flowering and fruit ripening, rainfall and humidity must be minimal during these phases. Rain during flowering reduces fruit set, while rain during ripening and pre-harvest increases the occurrence of fruit cracking, a physiological disorder that compromises cherry quality [64,65,66]. Our research group achieved significant results using net covers, which reduced the natural cracking index by 40% [67]. Additionally, we observed higher fruit weight values in trees covered with nets compared to the control group, with increases of 45% in 2019 and 13% in 2021. Altitude also influences fruit quality, with higher altitudes generally producing better-quality fruits due to more significant chilling accumulation. However, this relationship depends on the genotype of the cultivar, as different cultivars have varying altitudes and chilling requirements [68].

Soil properties are equally critical in ensuring cherry quality. Well-aerated and well-drained soil with a pH of between 6 and 6.8 and a depth of at least 1 m is essential for proper root development and efficient nutrient absorption [69]. Although cherry trees can tolerate various soil textures, from sandy loam to clay loam, adequate drainage is crucial. Soils with a pH of below 5.5 can lead to manganese and aluminum toxicity. In contrast, soils with a pH of above 8 can cause nutrient deficiencies in phosphorus, iron, zinc, and manganese, which hinder root growth and reduce tree vigor, ultimately affecting fruit quality [59]. Soil amendments for sweet cherry trees also influence their yield and fruit characteristics: Potassium is vital for carbon assimilation and metabolism regulation; affects fruit size, color, soluble solids, and acidity; and increases disease and cold resistance, while phosphorus is essential for cell membranes, energy transfer, enzyme activity, and photosynthesis, significantly contributing to fruit yield and the production of metabolites and soluble solids [70].

Considering the specific climatic conditions, selecting the appropriate cultivar for each region is imperative to ensure high-quality fruit production. Therefore, maintaining optimal soil conditions is crucial to ensure the quality and productivity of cherry fruits.

#### 3.1.2. Scion × Rootstock Interaction

Rootstock selection has gained significant importance in meeting fruit productivity and quality market demands. Various studies of our research group indicate the influence of the scion × rootstock combination on cherry quality [48,52]. Although fruit quality primarily depends on the genotype of the scion [52], it can also be influenced by the rootstock and edaphoclimatic conditions [71]. Hence, evaluating the impact of scion x rootstock on productivity and fruit quality is crucial. The choice of rootstock affects quality parameters such as fruit size, firmness, color, taste, soluble solid content, precocity, and resistance to cracking and diseases [72,73]. With the right combination of scion × rootstock, it is possible to obtain fruits with higher firmness, weight, sugars, vitamins, and phenolic compounds, thus enhancing the fruit’s antioxidant activity [48,74,75].

Jakobek et al. [76] found that chlorogenic, neochlorogenic, *p*-coumaric, and quercetin-*O*-rutinoside increased when cv. *Lapins* were grafted onto Piku 1 and Weiroot 13 rootstock, compared to F12/1, Gisela 5, Maxma 14, and Weiroot 158 rootstocks, while higher levels of total sugars were observed on F12/1 rootstock. A recent study [74] found that for cvs *Kordia* and *Carmen*, the clonal rootstocks Colt and Gisela 5 promoted the highest contents of phenolic compounds. At the same time, Oblacinska and M × M 14 induced the highest values in *Regina*. Another study by Usenick et al. [72] showed that cv. *Lapins* grafted onto Weiroot 72 rootstock had higher phenolic content, fruit weight, total soluble solid (TSS) content, and firmness. Prichko and Sivoplyasov [77] concluded that certain cultivar x rootstock combinations, such as ANT x Maaka 9-8, ANTD 12/20, S 33, Geghard, ANT x 2-77-1, ANT self-fertile 17, ANT w/n 5, and Chufut Kale, resulted in high-quality *Alexandria* cherries with a mass of over 10 g and improved taste and nutrient profiles.

#### 3.1.3. Orchard Management

Cherry trees, like other stone fruit trees, generally have low drought tolerance, particularly after fruit set, as during this period, the fruits rapidly increase in weight and diameter and demand more significant volumes of water [78]. Therefore, several studies aim to determine the most efficient irrigation system for achieving higher yields and better-quality cherries [78,79,80]. Water savings can be achieved in cherry orchards by implementing water management techniques such as regulated deficit irrigation (RDI) or partial rootzone drying (PRD). Carrasco-Benavides et al. [78] found that RDI strategies applied after harvest did not adversely affect fruit quality or yield and could achieve water savings of up to 20%. Further studies also show a reduced effect on fruit quality when irrigation was applied at 75% of crop evapotranspiration, with only changes in flesh firmness. In contrast, other parameters of quality (fruit weight, flesh/seed ratio, water-soluble solids (%), pH, titratable acidity (g/100 mL), and inverted and total sugars) were not affected [81]. Similar results were found by Houghton et al. [82], implying that irrigation water use can be reduced without affecting sweet cherry production or fruit quality, and by Yin et al. [83] using drip irrigation. Even so, there are differences in TSS, titratable acidity (TA), and maturity index (TSS/TA) when comparing fully irrigated trees (100% of their water needs before harvest); non-irrigated trees of cvs. *Lapins* and *Ambrunes* [84] have been found, and there is a higher total organic acid content in irrigated trees from cv. *Regina* [85] or TSS in cvs. *Cristalina* and *Skeena* [80]. These effects on sugar and acid content are fundamental, as these two components are responsible for the sensory quality of sweet cherries [86], influencing consumers’ acceptability.

Pruning to control crop load can also optimize fruit quality [87]. Several studies show that pruning can improve sweet cherry quality, depending on the pruning severity [88] and the time of pruning [89,90]. Comparing pruned with unpruned trees [91] shows increases in fruit firmness and variations in TSS and TA. Reducing crop load by pruning helps to balance the distribution of photoassimilates [87], increasing fruit quality [28].

Protection of trees with covers is expected to prevent damage caused by adverse environmental conditions [92] and reduce rain-induced fruit cracking, fruit damage from birds, and/or tree and fruit damage from hail on the cracking or change ripening date [93]. Using these cover systems can also change the quality traits of fruits. Indeed, some works point out lower TSS, TA, and firmness in fruits from covered trees than those without cover [94,95,96]. A lack of changes in sugar content [97,98] or fruit firmness [99] caused by covers hve also been recorded. The maturation time of cherries must also be considered. Schmitz-Eiberger et al. [100] observed that early-harvest cultivars grown in high tunnels exhibited similar TSS and firmness compared to non-covered control trees, while late-harvest cultivars showed a reduction in sugar content (10–30%) and firmness. The fruit’s position in the canopy can also reduce the effect of using covers [94,101]. In addition, different covers can result in diverse effects on TSS, TA, and firmness [102]. Further works indicate that fruits grow bigger when grown under cover [96], but these effects are cultivar dependent, with, for instance, no variations found for cvs. *Kordia* and *Regina*, while for cv. *Lapins*, an increase was found [97,98]. The effect of covers on fruit quality varies according to several factors. It is a crucial tool to support the decision-making by cherry growers and exporters, as they are determinants in the fruit acceptance process in destination markets [96,103].

Applying growth regulators or biostimulants can be an approach to modulating cherry quality. Biostimulants, like the protein-based glycine betaine, resulted in bigger fruits, with increased TSS content and lower TA in cv. *Staccato* [53], with similar results observed in cv. *Skeena* [104]. The use of algae-based products as biostimulants, namely, *Ascophyllum nodosum* or *Ecklonia maxima*, has also resulted in changes in fruit quality. Increases in weight [105,106], higher TSS, and lower acidity [53], as well as inverse behavior [107,108,109], have been found with the use of these biostimulants. Increases in TSS have also been found with the use of gibberellins [110], auxins [111,112], and tropical plant extract biostimulants [51]. Other biostimulants, like inorganic compounds or bacterial and fungal-based products, can influence fruit quality. For instance, using potassium silicate resulted in increased fruit firmness [103], while applying *Pseudomonas* BA-8 and *Bacillus* OSU-142 in cv. *Ziraat* resulted in increased fruit weight and TSS content [113,114]. Other plant growth regulators have improved sweet cherries’ quality in several ways. The use of calcium increased TSS [115,116], as did treatments with gibberellic acid, GA_3_ [117]. However, treatments of GA_3_ can also reduce the TSS content [118]. Fruit firmness can be improved by spraying GA_3_ [119,120,121], salicylic acid (SA), acetylsalicylic acid (ASA) [122], or methyl salicylate [123]. Bigger fruits have been recorded when applying GA_3_ [117,118,120,124], SA, ASA [122], oxalic acid [125], and methyl salicylate [123]. Moreover, it is important to note that the extensive use of pesticides in sweet cherry cultivation may lead to concerns about pesticide residues in the fruit, potentially altering the composition of bioactive compounds and necessitating careful management and monitoring [126].

### 3.2. Factors Influencing the Quality of Sweet Cherries During Postharvest

After harvest, cherries deteriorate quickly due to bruising of the skin, softening of the flesh, changes in the sugar–acid balance, loss of water, browning of the stem, and surface pitting [127,128]. Even so, adequate storage conditions or other strategies, like edible coatings, can help maintain quality for extended periods.

Temperature and relative humidity (RH) influence sweet cherry quality during post-harvest storage [129,130]. Harvest temperature is recommended to be between 10 and 20 °C [131], even though the suggested storage and transport temperature for cherries is 0 °C [21]. Hydrocooling or forced-air cooling usually eliminates heat from the field to storage [132]. Lowering fruit temperature immediately after harvest results in firmer fruits [133] with lower TSS content and with similar behavior for TA compared to fruits not refrigerated, regardless of the cultivar [134]. Hydrocooling has been proven to delay deterioration by reducing stem browning and surface shriveling and providing higher overall acceptability. However, no significant effect was found on external color and TSS content [133].

To improve the storage time of sweet cherries, temperature and RH must also be considered. A high RH is needed, in the 90–95% range [131,135]. This high RH can reduce water loss from fruits and stems, which would otherwise soften the fruit and stem browning [136]. The use of differentiated packaging systems can also help maintain the quality of sweet cherries in postharvest situations.

For instance, modified atmosphere packaging (MAP) is widely commercially used to extend the storage life of cherries [137]. MAPs change the package’s oxygen and carbon dioxide concentration, but several proportions of these gases have been suggested [135], from 0.03% CO_2_ to 0.5–2% O_2_ for cv. Bing [138] to 20% CO_2_ and 5% O_2_ for cv. Burlat cherries [139]. MAP delays physicochemical changes while helping to retain color by reducing oxidation and preventing water loss and fruit shriveling by maintaining a high-humidity environment [4,140,141,142]. Further works point out the positive effects of MAP in sweet cherry and stem color and firmness [142,143,144,145], while acidity usually drops considerably [140] and sugar content remains constant [139,146].

Another approach for maintaining cherry quality during storage is using edible coatings. These coatings have been used to extend the shelf life of fruits and vegetables, as they act as a semipermeable film to minimize the loss of water and slow the product respiration rate [147]. There are several types of edible coatings and films, depending on the substances that form the film/coating, the addition of plant extracts or other additives, and the mode of application [148]. Several coatings have been tested in sweet cherry, and positive results have been reported. These include guar gum and ginseng extract [149]; chitosan and alginate in combination with olive leaf extract [150]; calcium sulfate in combination with pomegranate peel extract [151]; alginate [152]; whey protein isolate, chitosan, and shellac [153]; locust bean gum, shellac, and beeswax [154]; *aloe vera* [155]; almond gum and gum arabic [156]; agacanth gum and eremurus extract [157]; and medicinal and aromatic plant extracts [55]. Even though different coatings present different properties and effectiveness, their use has been proven to maintain a longer shelf life, with reduced loss of quality of cherry fruits. For instance, sweet cherry fruits coated with chitosan + alginate + olive leaf extract exhibited reduced mass loss compared to control fruits [150], with similar results recorded using plant extracts [55]. These two works also show the positive effect of coating in maintaining fruit firmness. Maintenance of fruit quality parameters, namely, TA, TSS, color, and sensory profile, when compared with uncoated fruit, are also critical effects recorded for edible coatings, as has been extensively documented, e.g., [55,148,151,152,154,155].

## 4. Mechanisms of Action of Sweet Cherry Nutrients

Sweet cherries are a rich source of nutrients, possessing several essential vitamins and minerals that contribute to overall health. Among these, vitamin C, vitamin A, vitamin E, and an array of B vitamins play crucial roles in metabolic processes and disease prevention. Sweet cherry also provides essential minerals such as potassium, phosphorus, magnesium, calcium, iron, and manganese, which are crucial for maintaining bodily functions and preventing chronic illnesses. This section will explore the nutritional benefits of sweet cherry nutrients, highlighting their potential impact on health and well-being.

### 4.1. The Effects of Sweet Cherry Vitamins on Human Health

One of the major nutrients in sweet cherry is vitamin C (ascorbic acid), which primates cannot synthesize due to a lack of L-gulono-1,4-lactone oxidase, making fruits and vegetables their primary sources [158,159]. Despite the mechanisms of action not yet being fully understood, several roles have been attributed to vitamin C. One of these is a crucial cofactor for several enzymatic reactions, especially for metal ion-dependent enzymes, keeping their metal ions in a reduced state [160]. For instance, dioxygenases are essential in synthesizing carnitine, norepinephrine, serotonin, and collagen and are involved in histone demethylation [161]. Another attributed role of vitamin C is hormone synthesis, as it is essential for producing cortisol and adrenaline, making it extremely important in stress management and exercise recovery [158]. It is also recognized as having therapeutic roles in cancer, cardiovascular and neurodegenerative diseases, and infectious disorders [162,163]; however, the scarcity of rigorous clinical trials using this vitamin makes it challenging to draw definitive conclusions about the efficacy it has on these diseases [161]. Nonetheless, part of it can be attributed to its effective antioxidant potential, which aids cells under oxidative stress and cells with high metabolic activity, especially neurons [164,165]. Several studies have revealed that supplementation with vitamin C modulates the expression of oxidative stress genes while increasing the expression of genes related to DNA replication and repair [166,167].

Sweet cherry also possesses high amounts of vitamin A, primarily as β-carotene. Like other vitamins, this nutrient is also not synthesized by the human body, instead being acquired through the diet [168]. Several properties have been attributed to β-carotene, including antioxidant capacity, anti-inflammatory effect, and promotion of immune response [169]. Concerning its antioxidant properties, it has been observed that β-carotene can neutralize reactive oxygen species (ROS), reducing oxidative damage to the cells and DNA [170]. In fact, β-carotene has been observed to effectively reduce lipid peroxidation without causing genotoxic effects in several in vitro [171,172,173,174] and in vivo studies [175,176,177]. Another property attributed to β-carotene is the ability to protect gap junctional intercellular communication, which establishes cytoplasmatic communication between cells by reducing ROS-induced inhibition and promoting the expression of the connexin genes [178,179].

Another group of vitamins present in sweet cherries is vitamin E, mostly found as α-tocopherol. This vitamin has antioxidant properties, protecting cell membranes from oxidative damage. It has been associated with improved immune function and protection against neurological and cognitive diseases [180,181]. Moreover, other functions attributed to it include membrane stabilization and putative gene regulation [181].

B vitamins are also present in sweet cherry, namely, vitamins B3 (niacin) and B5 (pantothenic acid), which present higher concentrations, followed by B1 (thiamine), B2 (riboflavin), and B6 (pyridoxine), and in smaller amounts, B7 (biotin) and B9 (folate) [41]. B vitamins are usually involved in several different metabolic pathways, acting directly or being co-factors of other enzymes. Vitamin B3, also known as niacin, is essential for cellular metabolism, being a component of NAD and NADP, which are active participants in oxidation–reduction reactions [182]. Supplementation with this vitamin has been observed to reduce oxidative stress, confer neuroprotection [182,183], and alleviate the symptoms of dermatological diseases [184]. Therefore, the lack of niacin in the diet is correlated to conditions such as pellagra and mental diseases, being sometimes associated with several types of cancer [185]. Vitamin B5, pantothenic acid, is a significant component of co-enzyme A, making it essential in metabolizing carbohydrates and fatty acids, and in protein and energy production [186]. Similar to other B vitamins, a lack of pantothenic acid has been associated with neurodegeneration, with supplementation ameliorating these disease symptoms [186,187]. Thiamine, also known as vitamin B1, presents functions similar to those of the vitamins mentioned above. It has been anointed as a cofactor of several metabolic enzymes, including pyruvate dehydrogenases, a-ketoglutarate dehydrogenase, and cytosolic transketolase, which are involved in carbohydrate metabolism [188]. Thiamine deficiency results in reduced activity of these enzymes, which can lead to reductions in adenosine triphosphate (ATP) levels in several types of cells, leading to mitochondrial damage [188]. Among the effects of this deficiency are neurological problems due to reductions in the brain’s glucose metabolism, similar to what happens in patients with Alzheimer’s disease [189]. Vitamin B2 and riboflavin, identical to other vitamins, cannot be synthesized by humans and are acquired through the diet. Rarely deficient, this vitamin is essential as a precursor of coenzymes, DNA repair, energy metabolism, amino acid synthesis, and radical scavenger production [190]. As to the less common B vitamins present in sweet cherries, there is vitamin B6, known as pyridoxine, and vitamin B9, known as folate. Pyridoxine is related to several metabolic and physiological processes, especially in reactions related to amino acid synthesis, degradation, and neurotransmitter metabolism [191,192]. It is due to this that deficiency in vitamin B6 is usually observed in cases of epilepsy, as well as in peripheral neuropathy [192]. Nonetheless, it has also been associated with other diseases, including diabetes and heart problems [161]. Finally, vitamin B9, folate, is essential for DNA synthesis and repair. It has a prevalent role in preventing uracil incorporation and hypomethylation of DNA while also being required to maintain the methylation patterns in DNA [193]. This critical role in human health has been related to some cancers [194].

### 4.2. Sweet Cherry Essential Nutrients and Their Importance

Sweet cherry is also rich in several essential minerals, including potassium, phosphorus, magnesium, calcium, iron, and manganese [67]. Potassium is vital for maintaining optimal health, particularly in modern diets, as these are often deficient in this nutrient [195]. It is strongly associated with reducing blood pressure, lowering the risk of stroke and coronary heart disease, and protecting against age-related bone loss and kidney stone formation [196]. These benefits are more pronounced when potassium is consumed from natural sources like fruits, such as sweet cherries and vegetables, rather than supplements [195]. Another essential nutrient present in sweet cherries is phosphorus. This element is related to processes such as skeletal mineralization, metabolism of carbohydrates and fats, and cellular signaling [197]. Both low and high intakes of phosphorus in the diet can lead to severe bone conditions [197,198]. Moreover, it is highly correlated to calcium, another essential element in sweet cherries. Like phosphorus, calcium is necessary for bone health, muscle function, blood pressure, and cellular signaling [199]. Nonetheless, calcium deficiency is mainly related to chronic diseases connected to decreased bone health [200]. Magnesium is a co-factor of several enzymes involved in muscular processes, neuromuscular relations, and blood pressure regulation [201]. Furthermore, magnesium enables the active transport of calcium and potassium across cell membranes, making it essential for conducting nerve impulses, muscle contraction, maintaining vasomotor tone, and ensuring a normal heart rhythm [201,202,203]. Sweet cherry also possesses iron, another crucial element required for several biological processes, namely, oxygen transport, cellular respiration, and DNA synthesis [204]. A lack of iron in the system usually leads to anemia, which is especially common in developing countries, as it is more available in modern diets [205]. Finally, compared to the nutrients above, manganese is usually found in lower quantities in sweet cherries [67]. Nonetheless, manganese has been observed to play an essential role as a cofactor of several enzymes, including those involved in neurotransmitter synthesis and metabolism, brain development, and cellular homeostasis [206].

As mentioned, sweet cherries are a rich source of essential vitamins and minerals. These are crucial in various metabolic and physiological processes, including antioxidant defense, enzyme activation, hormone synthesis, and bone health. Therefore, consuming sweet cherries has several health benefits, including reducing the risk of chronic diseases, enhancing immune function, and improving overall health, making them a valuable addition to a balanced diet.

## 5. Sweet Cherries and Chronic Disease Management (Cardiovascular, Diabetes, and Neurodegenerative Conditions)

Numerous studies on sweet cherry extract’s antioxidant and anti-inflammatory properties [4,207] have been conducted to determine its potential in preventing and controlling chronic diseases, including cardiovascular diseases, diabetes, and neurodegenerative diseases [208]. Ridker [209] states in his study that the incidence of cardiovascular diseases increased considerably in patients with the highest quartile of C-reactive protein (CRP), a marker of inflammation, compared to those with a lower quartile. According to [5], increased oxidative stress and inflammation are among the leading causes of some inflammatory diseases, such as type 2 diabetes mellitus (DM2) and cardiovascular diseases (CVD). Results of epidemiological studies described by other researchers [210,211,212] indicate that the intake of fruit, namely, sweet cherries, cherry powder, and the indicated bioactive components from cherries, appears to have a protective effect against coronary heart disease by decreasing the production of ROS, namely, nitric oxide (NO) and lipid peroxidation [23,213].

Several studies recognize sweet cherries as a natural functional product due to their rich and diverse composition of bioactive compounds and health-promoting properties, primarily attributed to their antioxidant activity. The antioxidants in these fruits help combat oxidative stress, which is associated with premature aging and various chronic diseases, including cancer, cardiovascular diseases, and neurodegenerative conditions [48,207,212,214].

### 5.1. Cardiovascular Diseases

It is recognized worldwide that CVD is the leading cause of death in the world. An estimated 17.9 million people died from cardiovascular disease in 2019, representing 32% of all global deaths. Of these deaths, 85% were due to heart attack and stroke [215]. In the last twenty years, increased studies have investigated the various protective effects of polyphenolics present in the diet that increase protection against oxidative stress, the leading cause of CVD. Atherosclerosis (ATS), an inflammatory fibroproliferative disease caused by endothelial dysfunction, is the leading cause of CVD [216].

The interest in functional foods and nutraceuticals, that is, foods or parts that provide a concentrated biological component, which provides health benefits, whether in the prevention or treatment of diseases, has increased [217,218]. Regular consumption of fruits and vegetables is associated with reduced CVD risk factors, such as blood pressure, endothelial function, and low-density lipoprotein (LDL) cholesterol, all of which are crucial factors for heart health [4]. This is due to its composition being rich in polyphenols, which have important antioxidant and anti-inflammatory properties.

Several studies have been developed to show the health-promoting effects of sweet cherries. Xu et al. [219] and Xia et al. [220] observed the effect of reducing the impact of cardiovascular diseases in their studies on endothelial cells isolated from bovine arteries and on mouse foam cells, respectively, when exposed to the anthocyanin cyanidin-3-glucoside. Sweet cherry polyphenols are also associated with a decrease in serum triglycerides, total cholesterol, and liver triglycerides, and inhibition of the formation of fat cells [221,222]. Furthermore, the significance of cherry polyphenols in preventing risk factors for ATS has also been demonstrated, as inflammation and endothelial dysfunction have been reduced [223].

### 5.2. Diabetes

Several studies have shown evidence that sweet cherry promotes healthy glucose regulation. In fact, sweet cherry stimulates active glucose consumption by HepG2 cells (non-tumorigenic cells with high proliferation rates and an epithelial-like morphology that performs many differentiated hepatic functions) [4,224]. Moreover, the anti-inflammatory properties of sweet cherries may help manage conditions like diabetes by reducing markers of inflammation such as C-reactive protein (CRP) and interleukin-6 (IL-6) [207,225,226]. Some studies even suggest that sweet cherries may help regulate blood sugar levels, which is beneficial for preventing or managing diabetes. This effect is attributed to their low glycemic index and bioactive compounds that improve insulin sensitivity [227].

Oxidative stress plays a vital role in the pathogenesis of type 2 diabetes and its complications. Van der Werf et al. [227] determined, in vivo, the effects of 2-month-long cherry consumption in a high-fat/high-fructose (HFHF) model of diabetic rats. After two months of HFHF (high-fat diet “WESTERN RD” from Special Diets Services, Saint Gratien, France) added to 25% fructose in water as a beverage, male Wistar rats were divided into HFHF and HFHF enriched in cherry (nutritional approach) or standard diet ND (lifestyle measures) and ND plus cherry for two months. Metabolic, lipidic, and oxidative parameters were quantified. Tissue oxidative stress was assessed, and hepatic and vascular complications were characterized. Type 2 diabetes was induced after two months of the HFHF diet, characterized by systemic hyperglycemia, hyperinsulinemia, glucose intolerance, dyslipidemia, hyperleptinemia, and oxidative stress associated with endothelial dysfunction and hepatic complications. Two months of a cherry-supplemented diet, in addition to lifestyle measures, in rats with type 2 diabetes decreased and normalized the systemic disturbances, including oxidative stress complications. The authors concluded that cherry consumption normalized vascular function and controlled hepatic complications, thus reducing the risk of diabetic metabolic disorders and hepatic complications related to type 2 diabetes in rats.

Cherries can positively affect diabetes management due to their nutritional properties and beneficial phytochemicals [201,225]. The lower glycemic index of cherries relative to other fruits was observed by [228]. According to Gonçalves and collaborators [3], the anthocyanins in sweet cherries protect β cells against oxidative stress caused by glucose and complications associated with diabetes. This result agrees with findings by [229], who found that pancreatic α cells decreased glucagon production and increased hepatic glucose uptake and insulin production by pancreatic β cells. The same authors conclude that anthocyanins can reduce blood glucose levels by delaying glucose production from complex carbohydrates and hepatic glucose production.

In another study, a cell culture enriched with anthocyanins and anthocyanidins from sweet cherries showed a significant increase in insulin secretion [230].

Endothelin-1 (ET-1) plays a role in mitigating the harmful effects of diabetic vascular disease, as it is one of the vasodilators identified with important proliferative, profibrotic, and pro-inflammatory properties [231,232]. ET-1 is a pleiotropic molecule, and reducing its plasma concentrations through the consumption of cherries can lower the risk of various diseases, including diabetes [207].

An investigation into the effects of cherry anthocyanins on glycemic control was conducted using mice. These studies concluded that supplementary feeding of cherries reduced triglyceride synthesis, glucose, and leptin levels [233,234]. This result may be due to the fiber content of cherries [4].

Other studies have shown that the consumption of sweet cherries increased glucose uptake by cultured HepG2 cells [224]. Inhibition of the enzyme α-glucosidase, which is involved in the intestinal absorption of carbohydrates, was also observed. Chlorogenic acid, identified as one of the primary polyphenols in cherry juice, inhibited the enzymes α-glucosidase and dipeptidyl peptidase-4, which play a role in the onset of diabetes [3].

### 5.3. Neurodegenerative Diseases

Neurodegenerative diseases, such as Alzheimer’s, Parkinson’s, and amyotrophic lateral sclerosis, are characterized by the progressive degeneration and death of neurons in different regions of the nervous system. Due to its high oxygen consumption and lipid content, the brain is highly vulnerable to the effects of ROS. These diseases have a devastating impact on patient’s lives and represent a significant challenge for modern medicine. Recent studies have investigated the role of foods rich in antioxidants and bioactive compounds in the prevention and treatment of neurological diseases. Among these foods, cherries have attracted attention due to their antioxidant and anti-inflammatory properties [4,235,236]. The powerful antioxidant properties in cherries can protect brain cells from harmful effects and improve cognitive function due to their action, which neutralizes free radicals and reduces oxidative stress. Cyanidin 3-glucoside, for example, is one of the most clinically studied anthocyanins and can act as a neuroprotective agent [236,237]. The in vitro study by Matias et al. [235], using two different human cell lines, intestinal epithelial cells, and neuronal cells, investigated the application against cellular oxidative stress of an antioxidant extract rich in phenolics from Portuguese cherry (Saco cherry) residues. The extract was rich in anthocyanins, mainly cyanidin-3-rutinoside, cyanidin-3-glucoside, peonidin-3-glucoside, and neochlorogenic acid, and exhibited potent antioxidant chemical activity. Gonçalves et al. [1] and Serra et al. [238] also identified these compounds in other cultivars. The results showed that cherry extract efficiently prevented cell death induced by oxidative stress in neuronal cells. Therefore, given the results obtained, cherry extract could be a great option as a high-value ingredient for preventing disorders induced by oxidative stress or problems caused by intestinal inflammation or neurodegenerative diseases [235].

Cherries are rich in melatonin, a natural hormone that influences numerous physiological functions in the human body. Melatonin can cross the blood–brain barrier and potentially protect against oxidative damage in brain cells. As a result, this hormone possesses chronobiotic and sleep-inducing properties. Additionally, it has neuroprotective and antioxidant characteristics and helps with pain management [239]. Studies assessing melatonin concentration in cherries conducted by González-Gómez et al. [240] also observed that melatonin could have antioxidant and protective effects against oxidative stress. They concluded that the concentration of this hormone varies depending on the ripeness of the cherries. Zhao et al. [241] identified tryptophan decarboxylase (PaTDC), an enzyme in sweet cherries’ melatonin production. Furthermore, the same authors found that the expression of PaTDC is directly related to the level of melatonin produced in cherries.

In vitro studies conducted by Filaferro et al. [242] using an extract rich in cherry anthocyanins against Parkinson’s disease in a cellular model concluded that this extract has significant potential for formulating a nutraceutical product to prevent the effects of oxidative stress and related neurodegenerative diseases.

New sweet cherry cultivars (Sweet Aryana^®^ PA1UNIBO*, Sweet Lorenz^®^ PA2UNIBO*, Sweet Gabriel^®^ PA3UNIBO*, Sweet Valina^®^ PA4UNIBO*, Sweet Saretta^®^ PA5UNIBO*, Marysa^®^ PA6UNIBO* grafted on Colt, and Sweet Stephany^®^ PA7UNIBO* grafted on CAB11E) were tested to investigate their antioxidant and neuroprotective properties in SH-SY5Y cells, which are similar to neurons. The results suggest that these new cultivars are more effective in combating oxidative stress and positively regulating brain-derived neurotrophic factors, leading the authors to conclude that these new cultivars provide a pleiotropic intervention in combating neurodegeneration. However, in vivo studies are necessary to obtain more robust results. The authors also note that these findings open the door to improved selection in future breeding programs [12].

## 6. Sweet Cherries: Supporting Gastrointestinal Health

Over millions of years, the human digestive tract, along with those of other animals, has coevolved with a diverse community of microorganisms known as the gut microbiota. This coevolution has been mutually beneficial for both hosts and microorganisms: The microbiota aids in nutrient digestion, influences behavior, and regulates immune system activity. Some bacterial groups in the gastrointestinal tract have become specialized in surviving on host-derived compounds, while others rely more on dietary-derived compounds [243]. Nutraceuticals and functional foods have received significant interest due to their presumed safety and potential nutritional and therapeutic benefits. Consuming plant-derived products has been shown to play a crucial role in reducing physiological threats, thanks to the mechanisms of action of their chemical compounds, which enhance immune responses and the body’s defense system [244]. Red fruits, including cherries, are a significant source of antioxidants in the human diet, primarily due to their content of polyphenols such as flavonoids and anthocyanins, which offer numerous health benefits [244]. However, the positive effects of these bioactive phytocompounds are strongly associated with their bioavailability, which is reported to be low [245]. The stability of anthocyanins during gastrointestinal digestion is crucial for assessing their bioavailability. Consuming cherries can be a significant way to incorporate anthocyanins into the diet, potentially offering health benefits at the gastric level. Additionally, their degradation products and metabolites may provide antioxidant effects in the small intestine [245].

Several epidemiological studies have highlighted the significant role of polyphenols from fruits in preventing diseases such as cancer, metabolic disorders, cardiovascular conditions, and gastrointestinal issues [246]. However, after ingestion, these molecules are degraded in the gastrointestinal tract before reaching the bloodstream, where they exert their effects [244]. In simulated digestion, polyphenols are gradually released from the oral phase through to the gastrointestinal phases, with partial decomposition occurring in the small intestine due to instability. In contrast, fiber-bound phenols are released during colonic fermentation, producing high bioaccessible antioxidant activity [247]. Various studies using different methodologies and approaches have explored the bioaccessibility and bioavailability of sweet cherry compounds, showing that these compounds circulate in human blood as intact or metabolized conjugates [23,248]. More than 8000 phenolic compounds have been identified and extensively studied for their medicinal properties. Phenolics from various plant sources and at different ripeness levels can exhibit significantly different digestive patterns in the human gastrointestinal tract. Additionally, after consumption, only a tiny percentage (10–30%) of polyphenols from fruits and vegetables remain stable, are released, and are absorbed in the stomach but are easily degradable in the small intestine mainly due to the high value of the pH [23,245,248]. The polyphenols, bound to dietary fibers of the plant cell wall, can bypass gastric and small intestinal digestion and reach the colon, where the colonic microbiome metabolizes them [247].

The gastrointestinal tract microbiota is a diverse community of microorganisms that play crucial roles in the biology of multicellular hosts. Factors such as genetics, age, physiological status, pathology, metabolic activity, water chemistry, temperature, location, and trophic level of the host can significantly impact the composition and function of the intestinal microbiota [249]. The intestinal microbiome influences human body weight, digestive capacity, infection resistance, and the synthesis of essential nutrients. It plays a significant role in gut health by maintaining barrier function, providing antioxidant protection, and regulating the immune system [246].

Several studies have utilized in vitro methods to simulate and assess the effects of gastrointestinal digestion on phenolic compounds in fruits and their derivatives. While most of these studies have employed static methods, there is considerable variation in the parameters used and a lack of uniformity in the concepts of bioaccessibility, bioactivity, and bioavailability. This inconsistency complicates the accurate interpretation of digestion effects. Although efforts are being made to standardize these parameters, a consensus among researchers has not yet been reached. Therefore, future research must adopt standardized methods to better understand the changes in phenolics during gastrointestinal digestion [250]. However, there is limited information on how gastrointestinal digestion affects the bioaccessibility and bioactivities of phenolics in fruits, which could hinder further development, utilization, and enhancement of their economic value [251]. It is important to note that the total concentration of certain compounds in food does not always reflect how much the human body can absorb. When food is digested in the gastrointestinal tract, some bioactive compounds may be broken down or changed by digestive enzymes, affecting their effectiveness. Bioaccessibility refers to the amount of a compound that is released from food during gastrointestinal digestion and can be absorbed in the intestine. For bioactive compounds and micronutrients in food to be beneficial, they need to be released from the food during digestion and become available for absorption [252]. Therefore, it is crucial to determine the phenolic compound levels after gastrointestinal digestion rather than intrinsic contents and the effects of undigested matrix on human gut health [253].

Most studies on the gastrointestinal tract focus on fruit juices, with research directly involving whole fruits being limited, though some of these studies provide valuable insights. The kinetic approach developed by Dufour et al. [248] showed that polyphenol levels remain relatively stable for up to 150 min before gastric emptying. Additionally, consuming polyphenols at a nutritional dose through diced fruits and vegetables does not negatively impact protein digestibility. However, the intake of a phenolic extract might be detrimental to individuals with low protein intake. Therefore, it is essential to carefully consider the risk/benefit balance when consuming polyphenol supplements. Beconcini and collaborators [244] developed cherry nanoparticles that demonstrated the potential for extending the residence time of polyphenols in the gastrointestinal tract. This extension reduces the impact of intestinal clearance mechanisms and increases the available surface area for interaction with biological targets. Given that fresh cherry fruit is available only seasonally, nanoparticles formulated with protected thiolated chitosan enhance the encapsulated extract’s effectiveness and ensure that the fruit’s seasonal availability does not restrict the potential benefits of cherry consumption. The anthocyanin content in cherries is a crucial indicator of the bioprotective potential of cherry digestion, highlighting the importance of preserving these compounds to retain their protective benefits. Among some cherry cultivars, Sweetheart and Stella showed the highest bioprotective capacity, indicating that anthocyanin levels are better markers than vitamin C of a cultivar’s ability to protect human cells from oxidative stress [254].

## 7. Sweet Cherries and Their Potential in Cancer Prevention and Treatment

Cancer is characterized by the rapid growth, proliferation, and invasion of malignant cells into various tissues and organs. Although its causes are diverse and complex, our understanding of the disease remains incomplete [255]. Several epidemiological studies suggested that consuming vegetables and fruits may offer protection against cancer, largely due to their bioactive compounds [238]. Phenolic acids, flavan-3-ols, and anthocyanins, among other natural molecules, have been widely studied for their anti-inflammatory, antioxidant, and anti-mutagenic properties. These compounds contribute to cancer prevention by regulating cellular metabolism, inducing cell cycle arrest, inhibiting cell growth and differentiation, and modulating genes involved in tumor cell proliferation [9,256,257].

Sweet cherries are a rich source of phenolic compounds, including anthocyanins (mainly cyanidin-3-*O*-rutinoside), flavan-3-ols, ferulic acid, chlorogenic acids, gallic acid, quercetin, syringic acid, and *p*- and *m*-coumaric acids. The concentration of these compounds is influenced by factors such as genotype, fruit ripeness, climate, and storage conditions [1,9,238,257,258,259,260]. Numerous studies, both in the laboratory and in living organisms, have highlighted the anticancer properties of polyphenolic compounds, which may help prevent cancer by inhibiting various stages of tumor development, including initiation, promotion, and progression [261]. In particular, phenolic acids, flavan-3-ols, and anthocyanins have drawn significant attention for their potential anti-inflammatory, antioxidant, and anti-mutagenic properties [262]. Several studies have shown that anthocyanins in cherries, particularly cyanidins, can potentially slow tumor growth [263,264].

As oxidative stress and chronic inflammation are closely linked to carcinogenesis, the antioxidant and anti-inflammatory effects of polyphenols are promising for protecting against cancer and slowing its progression [265]. Research suggests that these compounds may interfere with cancer development by modulating cellular processes such as metabolism, cell cycle regulation, and gene expression associated with tumor growth [9,256]. Their enhanced antioxidant properties further contribute to their anti-mutagenic potential, as they help reduce ROS levels, which can impact tumor cell development [266,267]. Additionally, phenolic compounds can reduce oxidative stress by countering the ROS produced by tumor cells, thereby influencing various stages of carcinogenesis and processes like tumor proliferation.

Studies have shown numerous health benefits from consuming cherries, including antioxidant effects [2,238,257] and anticancer activity against human colon cancer cells (HT-29 and HCT-15) and stomach cancer cells (MKN45) [238]. As a result, sweet cherries are recognized for their anticancer properties [4]. Moreover, they exhibit specific effects such as cell cycle arrest, induction of apoptosis, inhibition of oxidative stress-induced DNA damage, suppression of angiogenesis, and activation of detoxification enzymes (phase II enzymes) [4]. Sweet cherries have also been found to reduce cell growth by inhibiting the activation of protein kinase B (Akt) and phospholipase C gamma 1 (PLCγ-1), leading to a higher Bax/Bcl-2 ratio and triggering both intrinsic and extrinsic apoptotic processes [268]. Furthermore, the anthocyanins in sweet cherries display a chemotherapeutic effect by downregulating the mRNA expression of several metastatic biomarkers, such as Sp1, Sp4, and vascular cell adhesion molecule 1 [269]. The most well-documented biological effects of sweet cherry extract are its antioxidant and anti-inflammatory properties [26,207]. Table 1 summarizes the health effects of cherry consumption and related products on human health.

### 7.1. Anti-Inflammatory Potential

Inflammation is the body’s natural defense mechanism against microbial infection or tissue injuries, aiming to eliminate, neutralize, or destroy harmful stimuli [270]. However, excessive inflammation can be harmful to tissues and act as a precursor to various disorders, including cancer [271,272]. The anti-inflammatory properties of sweet cherries are well documented, with multiple studies highlighting their potential to inhibit key inflammatory markers such as cyclooxygenases (COX-1 and COX-2), prostaglandin E2 (PDE2), interleukin 6 (IL-6), tumor necrosis factor-alpha (TNF-α), endothelin-1, and plasminogen activator inhibitor-1. This inhibitory effect is also linked to the downregulation of mitogen-activated protein kinase (MAPK) and the upregulation of interleukin2 (IL-2) and interleukin 4 (IL-4) [43,225].

Key pathways targeted by flavonoids include the activation of molecules involved in cell proliferation and death, such as tumor suppressor p53 (cellular tumor antigen), which triggers a cascade of events leading to apoptosis. This underlies flavonoids’ anti-proliferative effects. Additionally, the Akt/FAK/Ras/PI3K pathway, known for its role in malignant progression, contributes to the anti-metastatic effects of flavonoids. The MAPK pathway, another important target, plays a role in anti-inflammatory responses [273]. Phosphorylation of MAPK family members extracellular-signal-regulated kinase 1/2 (ERK1/2) and p38 has been observed, with cherry anthocyanin-rich fractions inducing early and sustained activation of phospho-ERK1/2. Overall, these fractions exhibited greater potency by simultaneously upregulating both phospho-ERK1/2 and phospho-p38. Moreover, phenolic compounds from sweet cherries suppressed the growth of MDA-MB-453 breast cancer cells by activating cell signaling pathways that promote apoptosis and inhibit cell invasion, with anthocyanin-enriched fractions demonstrating stronger chemopreventive effects [268].

The anti-inflammatory potential of cherries is largely attributed to the presence of anthocyanins, which have shown significant inhibition of COX-2 [274,275]. This is a notable finding, as COX2 is a major source of prostaglandins in cancer cases, suggesting a possible anti-cancer role of sweet cherries [276]. This regulatory effect is believed to result from phenolics’ ability to downregulate nuclear factor-kappa B (NF-κB), which influences the biosynthesis of inducible nitric oxide synthase (iNOS) and COX-2. Consequently, this downregulation reduces the production of nitric oxide (NO) and prostaglandins while also suppressing mitogen-activated protein kinases (MAPKs) and JNK1/phosphorylation [277].

Beyond anthocyanins, other phenolic compounds in cherries, such as hydroxybenzoic acids, caffeic acid, *p*-coumaric acid, and quercetin, have also shown effectiveness in modulating inflammation-related pathways and reducing pro-inflammatory markers [278,279]. The anti-inflammatory effects of consuming sweet cherries have been observed in humans as well. In one study involving 20 healthy participants, daily consumption of 280 g of sweet cherries for 28 days led to reduced levels of C-reactive protein (CRP) and NO [226]. Another study with 18 healthy participants found that 280 g of daily sweet cherry consumption over 28 days resulted in lower levels of CRP, epidermal growth factor (EGF), endothelin-1 (ET-1), extracellular receptor for advanced glycation end-product binding protein (EN-RAGE), ferritin, IL-18, and plasminogen activator inhibitor-1 (PAI-1). Additionally, the expression of IL-1 receptor antagonist increased [225].

Participants were assessed 28 days after the intervention to evaluate the persistence of these anti-inflammatory effects. While ferritin levels continued to decrease and CRP levels remained low, the other biomarkers either completely or partially returned to their baseline levels [225].

### 7.2. Antioxidative Properties

The generation of ROS, including hydrogen peroxide (H_2_O_2_), hydroxyl radicals, and reactive nitrogen species, plays a vital role in maintaining cellular function and tissue homeostasis [266,267]. However, persistent oxidative stress (OS) and moderate ROS levels have been linked to tumor initiation, growth, progression, and aggressiveness [280,281]. Interestingly, inducing extreme OS to trigger programmed cell death in tumors has emerged as a potential anti-cancer therapy [282,283].

On the other hand, the antioxidant properties of natural bioactive compounds have proven effective in reducing moderate OS levels and thereby the processes that contribute to cancer development and progression. Multiple studies have demonstrated that sweet cherries possess a high capacity for scavenging free radicals [10,258,284]. The potent antioxidant capacity of sweet cherries is largely influenced by their rich composition of bioactive compounds [238,258]. Among these compounds, anthocyanins, particularly cyanidin-3-*O*-rutinoside and cyanidin-3-*O*-glucoside, play a crucial role in sweet cherries’ high antioxidant potential [238,254].

The antioxidant effects of sweet cherries have been observed in Caco-2 cells, which have been attributed to their anthocyanin content [238,258]. In addition to anthocyanins, other phenolic compounds, such as *p*-coumaroylquinic acid, along with various flavanols and flavonoids, also contribute to the antioxidant properties of sweet cherries [238].

Research suggests that the consumption of antioxidants, measured as total radical-trapping antioxidant potential, is linked to a reduced risk of both cardia and distal stomach cancers [285]. Moreover, a higher dietary intake of certain flavonoid categories is associated with a lower risk of colorectal cancer [286]. The health benefits of sweet cherry consumption have been demonstrated in several studies. For example, Prior et al. [287] found that eating 280 g of sweet cherries daily for six consecutive days significantly increased plasma lipophilic and hydrophilic antioxidant capacity.

To combat postprandial oxidative stress, it is recommended to include foods rich in antioxidants with every meal [287]. Cherry consumption has been shown to improve various markers of antioxidant capacity, such as increasing plasma oxygen radical absorbing capacity (ORAC) levels [288] and total serum antioxidant status [289], while also reducing lipid peroxidation [290].

### 7.3. Anticarcinogenic Activity

Research has shown that consuming sweet cherries may reduce cancer risk [291]. Lin et al. [292] demonstrated that anthocyanins in sweet cherry significantly inhibit tumor growth, invasion, and metastasis. In particular, cyanidin-3-*O*-glucoside exhibits anticancer effects through various mechanisms [293], including antimutagenic proprieties [294], cell cycle arrest [295], induction of apoptosis [296], and inhibition of angiogenesis [297]. Peonidin-3-*O*-glucoside, cyanidin-3-*O*-glucoside, and cyanidin-3-*O*-rutinoside, the predominant anthocyanins in sweet cherries, exhibit strong anti-invasive properties, significantly reducing the invasion of A549 cells [298].

Cyanidin-3-*O*-glucoside notably suppresses cell growth by inducing G2/M arrest, which is linked to decreased levels of cyclin-dependent kinase (CDK)-1, CDK-2, cyclin B1, and cyclin D1, along with increased caspase-3 activation, chromatin condensation, and cell death [299]. Furthermore, cell lines treated with sweet cherry anthocyanins showed reduced proliferation and increased apoptosis [299]. Cyanidin may also lower the risk of malignant transformation by promoting cellular differentiation [300]. Studies that have investigated the anticancer effects of sweet cherry extract on human prostate cells revealed decreased viability in both neoplastic and non-neoplastic cell lines [301]. Additionally, sweet cherry anthocyanins regulate cellular metabolism and induce cell cycle arrest.

**Table 1:** Summary of the health effects of cherry consumption and associated products on human health.

Product/Compound	Disease	Effect	References
Sweet cherries, cherry powder	Cardiovascular	Protective effect against coronary heart disease by decreasing the production of ROS, namely, nitric oxide (NO) and lipid peroxidation.	[23,213]
Cyanidin-3-*O*-glucoside	Cardiovascular	Cardioprotective: improving the function of endothelial cells isolated from bovine arteries and reducing the formation of foam cells in mice.	[23,213]
Sweet cherry polyphenols	Cardiovascular	Din serum triglycerides, total cholesterol, liver triglycerides, and inhibition of the formation of fat cells.	[23,213]
Cherry polyphenols	Cardiovascular	Preventive effect against risk factors for atherosclerosis (ATS), showing reduced inflammation and improved endothelial dysfunction.	[23,213]
Sweet cherry	Diabetes	Promotes active glucose consumption by HepG2 cells.	[4,224]
Sweet cherry	Diabetes	Reduced markers of inflammation such as C-reactive protein (CRP) and interleukin-6 (IL-6).	[207,225,226]
Cherry-supplemented diet	Diabetes	Decreased and normalized the systemic disturbances, including oxidative stress complications.	[23,213]
Cherry anthocyanins	Diabetes	Protect the β cells against oxidative stress caused by glucose and complications associated with diabetes.	[23,213]
Anthocyanins	Diabetes	Reduced blood glucose levels by delaying glucose production from complex carbohydrates and hepatic glucose production.	[23,213]
Cell culture enriched with anthocyanins and anthocyanidins from sweet cherries	Diabetes	Showed a significant increase in insulin secretion.	[23,213]
Supplementary feeding of cherries on mice	Diabetes	Reduced triglyceride synthesis, glucose, and leptin levels.	[23,213]
Sweet cherries	Diabetes	Increased glucose uptake by cultured HepG2 cells. An inhibition of the enzyme α-glucosidase was also observed.	[23,213]
Chlorogenic acid in cherry juice	Diabetes	Inhibited the enzymes α-glucosidase and dipeptidyl peptidase-4.	[23,213]
Cyanidin-3-*O*-glucoside	Neurodegenerative	Neuroprotective agent.	[236,237]
Cherry extract	Neurodegenerative	Efficient in the prevention of cell death induced by oxidative stress in neuronal cells.	[23,213]
Cherry melatonin	Neurodegenerative	Potential to protect against oxidative damage in brain cells.	[23,213]
Extract rich in cherry anthocyanins	Neurodegenerative	Potential for formulating a nutraceutical product to prevent the effects of oxidative stress and related neurodegenerative diseases.	[242]
New cherry cultivars	Neurodegenerative	More effective in combating oxidative stress and positively regulating brain-derived neurotrophic factors, and combating neurodegeneration.	[12]
Cherry anthocyanins	Gastrointestinal	Bioprotective potential of cherry digestion.	[254]
Phenolic acids, flavan-3-ols, and anthocyanins	Cancer	Cancer prevention by regulating cellular metabolism, inducing cell cycle arrest, inhibiting cellular growth and differentiation, and controlling genes associated with tumor cell proliferation.	[9,256,257]
Phenolic acids, flavan-3-ols, and anthocyanins	Cancer	Anti-inflammatory, antioxidant, and anti-mutagenic properties.	[262]
Cherry anthocyanins	Cancer	Potential to impede tumor growth.	[263,264]
Sweet cherries	Cancer	Anticancer activity against human colon cancer cells (HT-29 and HCT-15) as well as stomach cancer cells (MKN45).	[238]
Sweet cherries	Cancer	Anticancer effects such as cell cycle arrest, induction of apoptosis, inhibition of oxidative stress-induced DNA damage, suppression of angiogenesis, and activation of detoxification enzymes (phase II enzymes).	[4]
Sweet cherries	Cancer	Reduced cellular growth by suppressing protein kinase B (Akt) and phospholipase C gamma 1 (PLCγ-1) activation, leading to an enhanced Bax/Bcl-2 ratio and subsequently activating intrinsic and extrinsic apoptotic processes.	[268]
Sweet cherries anthocyanins	Cancer	Exhibited a chemotherapeutic effect by downregulating the mRNA expression of several metastatic biomarkers, including Sp1, Sp4, and vascular cell adhesion molecule 1.	[269]
Cherry anthocyanins	Cancer	Demonstrated significant inhibition of COX-2, suggesting a potential anti-cancer role.	[273,274,275]
Sweet cherry anthocyanins	Cancer	Had a significant role in inhibiting tumor growth, invasion, and metastasis.	[292]

## 8. Enhancing Wellness: Sweet Cherries and Exercise Recovery

Regarding exercise recovery, cherries, particularly cherry juice, have become a central element in athlete’s recovery strategies and are now commonly used to enhance wellness. The physiological benefits of cherry and cherry juice consumption have primarily been studied in the context of sports nutrition for muscle recovery. Although this assumption remains somewhat controversial, numerous studies over the last decade have explored this area [25]. According to McHugh [302], the limited use of sweet cherry juice for recovery after muscle fatigue is due to its higher cost and lower availability compared to tart cherry juice, rather than any significant differences in phenolic concentrations between them. Both types of cherries exhibit similar antioxidant and anti-inflammatory properties, which help reduce muscle soreness and improve overall recovery time.

In a review of the health benefits of cherries, Kelley et al. [207] found that eight out of nine studies showed a significant reduction in exercise-induced muscle pain, soreness, and strength loss after cherry consumption. To clarify and better understand the results of various parameters evaluated in exercise recovery studies, McHugh [302] introduced “precovery” to describe interventions initiated in the days leading up to exercise to facilitate recovery in the days after exercise. This term is preferable over “recovery,” which refers to intervention introduced after exercise, as studies have consistently shown that muscle function recovers faster when cherry juice is consumed several days before exercise. The available evidence does not support starting a regimen on the day of or after exercise. Instead, it supports the use of cherry juice as a “precovery” strategy across various athletic activities [302].

## 9. Holistic Impact: Metabolic, Weight, and Skin Health Effects of Sweet Cherries

**Figure 2:**
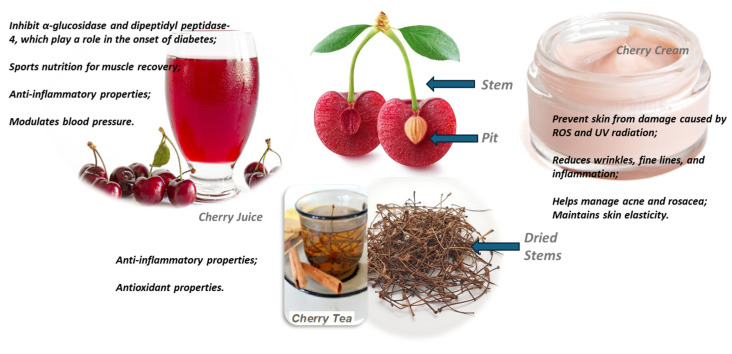
An approach embracing a holistic view of cherry consumption, considering cherry by-products, sustainability, metabolism, weight management, and skin health [4].

A holistic approach to cherry consumption (Figure 2) involves considering the various aspects of health and wellness that cherries can influence, incorporating them into a balanced diet, and understanding their benefits beyond just nutrition. Cherries can be consumed fresh, dried, as juice, or in supplement form. Each form offers different benefits and can be chosen based on dietary preferences and needs. Fresh or dried cherries can be used in salads, yogurt, or oatmeal, or as a topping for desserts. For a sweet–tart flavor, incorporate cherries into savory dishes like sauces for meats or grain bowls. Cherry juice can be consumed as a refreshing drink, added to smoothies, or used as a base for sauces and marinades.

Sweet cherries are delicious and have various health benefits. Their effects on metabolic health, weight management, and skin health are noteworthy [4]. Let us investigate each of these areas in detail.

### 9.1. Metabolic Health and Weight Management

Dark sweet cherries are rich in fiber and polyphenols, which help reduce risk factors associated with obesity. In a single-blind, randomized, placebo-controlled study, Arbizu et al. [50] investigated the effects of dark sweet cherries’ on inflammation, cardiometabolic health, and liver health biomarkers in obese adults. The participants (40 adults with body mass index (BMI) = 30–40 kg/m^2^) consumed 200 mL of juice supplemented with sweet cherry powder or a placebo drink twice/day for 30 days. Anthropometric and physiological biomarkers were monitored on day 1, day 15, and at the study endpoint (day 30). Blood inflammatory biomarkers were assessed at all points of the study, and blood lipids, glucose, and liver enzymes were measured at the beginning and end of the study. Individuals who consumed the supplemented juice showed lower systolic blood pressure (SBP) and a decrease in diastolic blood pressure (DBP) compared to the placebo group. A more significant SBP reduction was found in participants with a BMI > 35. Moreover, lower pro-inflammatory interferon-gamma (IFNγ) was correlated with SBP changes. The study concluded that dark sweet cherries helped decrease blood pressure levels and inflammation in obese adults.

Regular consumption of sweet cherries has also been linked to improvements in lipid profiles, including reductions in total cholesterol, low-density lipoprotein (LDL) cholesterol, and triglycerides [226,303]. It is important to note that some studies present different results. Eslami et al. [304] found no significant effects of cherry consumption on glycemic markers and lipid profile. The authors performed four database searches up to June 2021. Randomized controlled trials investigating the effects of tart or sweet cherries on glycemic markers (fasting blood glucose, fasting insulin, and HOMA-IR—Homeostatic Model Assessment for Insulin Resistance, where a score greater than 1.9 indicates early insulin resistance and greater than 2.9 indicates significant insulin resistance) and serum lipids (triglycerides, total cholesterol, LDL–cholesterol, and high-density lipoprotein (HDL)–cholesterol) were included in the meta-analysis. The results showed no significant effect of cherry consumption on glycemic markers or serum lipids. The authors noted methodological weaknesses in the studies. They recommended further research with larger samples and longer follow-ups to provide more rigorous evidence on the health effects of cherries, particularly in populations with abnormal metabolic profiles.

### 9.2. Skin Health Effects

In the cosmetics industry, there is a constant demand for new and innovative ingredients for product development. Both cosmetic companies and consumers are particularly interested in compounds derived from natural sources due to their numerous benefits. In a study by Agulló-Chazarra et al. [305], new and environmentally friendly extraction techniques—such as pressurized solvent, supercritical CO_2_, and subcritical water extractions—were used to obtain three new extracts from sweet cherry stems, a byproduct generated by the food industry. The study identified 57 compounds, including flavonoids, organic and phenolic acids, fatty acids, and terpenes, in these extracts. The extract obtained with supercritical CO_2_ exhibited the best characteristics, including a vast antioxidant capacity, especially against lipid peroxyl and •OH free radicals, and notable photoprotective and anti-aging properties. This makes it a potential new ingredient to be considered in developing new cosmetics.

Additionally, the industrial processing of cherries generates byproducts such as cherry pits, which can contribute to environmental concerns if not properly managed. Decot et al. [306] investigated the potential of cherry pits as antioxidants for possible use in cosmeceutical applications, aiming to find a sustainable strategy to minimize the ecological impact of cherry processing. Their study used eco-friendly techniques to perform metabolomic analyses of cherry pit extracts in water and a water–ethanol mixture, revealing enrichment in coumaroyl derivatives and flavonoids. The antioxidant activity of the extracts was tested on human skin cells exposed to H_2_O_2_ or LPS (lipopolysaccharide), simulating environmental oxidative stress. Both cherry pit extracts effectively reduced ROS from H_2_O_2_ or LPS without harming cell viability. Moreover, the extracts increased the expression of antioxidant enzymes like catalase and superoxide dismutase (SOD1), while decreasing the expression of nitric oxide synthase (NOS2), a pro-oxidant regulator. These findings highlight the antioxidant properties of cherry pits, suggesting their potential as a natural ingredient in skincare products and offering a sustainable solution for reducing food byproduct waste.

Sweet cherries are also enriched with melatonin, as reported by [3]. Melatonin helps regulate sleep and has antioxidant properties that protect skin cells from damage and improve skin health.

Due to all these properties, sweet cherries’ compounds may help prevent skin from damage caused by free radicals and UV radiation, reducing signs of aging like wrinkles and fine lines and reducing inflammation; sweet cherries can help manage skin conditions such as acne and rosacea. They also aid in soothing irritated skin. Moreover, sweet cherries’ high water content helps keep the skin hydrated, maintains elasticity, and prevents dryness.

Moreover, mindfulness and sustainability are important factors to consider: Eating cherries when they are in season and locally sourced can enhance their flavor and nutritional value while reducing environmental impact; choosing organic cherries can minimize pesticide exposure and support sustainable farming practices. Additionally, enjoying cherries mindfully by savoring their taste and texture can enrich eating and promote a healthy relationship with food.

## 10. Final Remarks

In conclusion, this review has comprehensively examined the diverse and significant health benefits of sweet cherries’ nutrients, from their cultivation to their role in human wellness. Sweet cherries (*Prunus avium* L.) are a rich source of bioactive compounds, including polyphenols, carotenoids, and essential vitamins such as vitamin C, which are crucial in combating oxidative stress and inflammation. These nutrients collectively help reduce the risk of chronic diseases, including cardiovascular disorders, diabetes, neurodegenerative conditions, and potentially even cancer.

While the research synthesized highlights the promising health-promoting properties of sweet cherries, it is important to acknowledge several limitations in the existing studies. There is considerable variability in study designs and sample sizes, which makes it difficult to generalize findings across populations. Furthermore, much of the evidence is derived from short-term studies, emphasizing the need for more extensive clinical trials to confirm the long-term health benefits of sweet cherry consumption. The mechanisms of action of the bioactive compounds found in sweet cherries also require further exploration, particularly in diverse populations and specific health conditions.

Despite these limitations, advanced crop techniques play a critical role in optimizing the beneficial compounds in sweet cherries, enhancing their potential impact on human health. The wide array of bioactive nutrients in sweet cherries supports various physiological functions, including improved gastrointestinal health, enhanced exercise recovery, better sleep modulation, and support for metabolic health, weight management, and skin health.

The findings provide a groundwork for further exploration of sweet cherries’ therapeutic potential, providing valuable insights for researchers and healthcare professionals. Consumers are encouraged to include these nutrient-dense fruits in their diets, as sweet cherries’ antioxidants and anti-inflammatory compounds make them an excellent choice for boosting overall nutritional intake and supporting health.

When incorporated into a balanced diet and complemented by regular physical activity, sweet cherries can significantly enhance overall health and well-being. As our understanding of these benefits continues to grow, sweet cherries stand out not only as a delicious fruit but also as a valuable component of a health-promoting diet.

## Data Availability

Not applicable.
